# The London Panel Committee Meeting

**Published:** 1915-02-27

**Authors:** 


### The London Panel Committee Meeting.

CONDITIONS OF SERVICE; THE NEW SYSTEM OF CERTIFICATION.

At a meeting of the London Panel Committee last Monday, the first resolution to be passed was one expressing sympathy with the relatives of the late Dr. H. J. Richards, who was Chairman of the Committee last year.

### Criticisms of the Medical Service.

The General Purposes Sub-Committee stated that, in view of the criticisms made from time to time as to the adequacy of the medical service in various areas in London, and the suggestions put forward for radical alterations in the conditions of service, it was desirable that the Panel Committee should prepare statistics with regard to practitioners' work. At present there did not appear to be available in an accessible form reliable information as to the amount of work actually being performed by practitioners on the panel, nor as to the time they were devoting to that work. As the information could only be obtained from the practitioners themselves, a schedule of questions had been prepared, and the Sub-Committee proposed to send these to practitioners.

The suggestion aroused some criticism. Dr. H. G. Cowie urged that the adequacy of treatment rendered by an individual practitioner depended upon personal factors, which could not be estimated by inquiry. Dr. Fletcher thought the number on the lists was the principal factor, but Dr. Cunnington suggested that if 2,000 was a suitable list in a poor neighbourhood, where the incidence of sickness was high, the limit might well be fixed at 3,000 in a district having a well-to-do population. Dr. Toland thought that an inquisition of the character proposed would be most derogatory. The best check upon the efficiency of the practitioner was the exercise by the insured person of his right to choose another doctor. To send a set of questions to practitioners was impertinent. Dr. Bernstein urged that the adequacy of the pharmaceutical service should also be considered.

Dr. B. A. Richmond (Secretary of the Committee) said the information was principally required for the protection of practitioners themselves. There was much criticism of the present system, and when an attack was made practitioners should have material with which to defend themselves. Dr. Atteridge said he believed the inquiry would show that the industrial classes were receiving very much better treatment than they were before the Insurance Act was passed.

### The New System of Certification.

The Panel Service Sub-Committee invited the Committee to express the opinion that books of certificate forms should be supplied to practitioners not on the panel, so that they might be able to give certificates in the form required by approved societies.

This led to a debate on the general question of certification, and adverse comments were made as to the practice of some approved societies of requiring a weekly certificate in chronic cases, and of refusing to pay sick benefit except up to the date of the last certificate. The Chairman (Dr. H. J. Cardale) remarked that the new system of certification was the result of concessions on the part of both doctors and approved societies, but some approved societies were interpreting the new rules in a way which made them a fresh burden instead of a benefit to the doctors. Practitioners were loyally observing their side of the bargain, but approved societies were taking advantage. Ultimately the Sub-Committee took back its report with a view to representations being made to the Commissioners on the general working of the new system of certification.

### The Number of Panel Practitioners.

The Panel Service Sub-Committee noted that the medical list for 1915 contained the names of 1,449 practitioners in general practice and the names of 103 institutions whose medical officers were under agreement to treat insured persons. In this connection the Sub-Committee remarked that it was reported to the British Medical Association last July that there were then resident in the county 1,303 practitioners on the panel, 969 practitioners not on the panel in general practice, and 2,957 practitioners not on the panel not in general practice.

